# Clinical and mutational profile of AT-rich interaction domain 1A-mutated cancers

**DOI:** 10.37349/etat.2023.00163

**Published:** 2023-08-31

**Authors:** Rosa Falcone, Marco Filetti, Pasquale Lombardi, Valeria Altamura, Francesco Paroni Sterbini, Giovanni Scambia, Gennaro Daniele

**Affiliations:** Istituto Nazionale Tumori-IRCCS-Fondazione G. Pascale, Italy; ^1^Phase 1 Unit, Fondazione Policlinico Universitario A. Gemelli IRCCS, 00168 Rome, Italy; ^2^Scientific Directorate, Fondazione Policlinico Universitario A. Gemelli IRCCS, 00168 Rome, Italy; ^3^Department of Life Science and Public Health, Università Cattolica del Sacro Cuore, 00168 Rome, Italy

**Keywords:** AT-rich interaction domain 1A, cancer, mutation, target

## Abstract

**Aim:**

AT-rich interaction domain 1A (*ARID1A*) encodes a key component of the SWItch/Sucrose Non-Fermentable (SWI/SNF) chromatin remodeling complex that participates in gene expression. *ARID1A *alterations are quite common among cancer patients, although their role remains debated. The aim of this article was to study ARID1A-mutated cancer patients.

**Methods:**

Molecular and clinical data of cancer patients evaluated at Phase 1 Unit of Fondazione Policlinico Universitario A. Gemelli IRCCS were collected. Molecular analyses were performed using FoundationOne^® ^CDx (Foundation Medicine Inc., Cambridge, MA, United States). Cancer patients with at least one molecular alteration in *ARID1A* gene were identified as ARID1A^+^.

**Results:**

Among the 270 patients undergoing molecular analysis, we found 25 (9%) with at least one pathogenic alteration in ARID1A. The vast majority of these patients were female (84%). The median age at diagnosis was 59; most of the cancers (15, 60%) were gynecological (especially endometrioid endometrial cancers and clear cell ovarian cancers), diagnosed at an early stage. Frameshift alterations in ARID1A were the most common (19/31, 61%) alterations. The median number of mutations in ARID1A^+^ population was higher compared to ARID1A^–^ population (6 *vs*. 4), as well as tumor mutational burden (TMB) [20 mutations/megabase (mut/Mb)* vs.* 1.26 mut/Mb]. Phosphatidylinositol-4,5-bisphosphate 3-kinase catalytic subunit alpha (*PIK3CA*), phosphatase and tensin homolog (*PTEN*), catenin beta 1 (*CTNNB1*), and lysine methyltransferase 2D (*MLL2*) mutations were enriched in ARID1A^+^ population. In this cohort, ARID1A did not display any relation with response to platinum chemotherapy. Cancers with double alterations in ARID1A (ARID1A^2+^) were all gynecological cancers (83% endometrioid endometrial cancers).

**Conclusions:**

This analysis provides clinical and molecular details about the phenotypes of ARID1A^+ ^cancers, in particular the subgroup of gynecologic cancers. The high frequency of concurrent mutations in the phosphoinositide 3-kinase (PI3K) pathway among endometrioid endometrial cancers may support the proposal of a new treatment strategy based on the combination of ataxia telangiectasia and Rad3-related (ATR) inhibitor and PIK3CA inhibitor.

## Introduction

AT-rich interaction domain 1A (*ARID1A*) gene, also known as Brahma-related associated factor 250a (BAF250a), is located on chromosome 1p36.11. It encodes a key component of the SWItch/Sucrose Non-Fermentable (SWI/SNF) chromatin remodeling complex [1]. This machinery, regulating chromatin architecture, controls gene expression, in particular, the transcription of those genes involved in oncogenesis, such as the phosphoinositide 3-kinase (PI3K)/protein kinase B (AKT)/mammalian target of rapamycin (mTOR) pathway and steroid receptor signaling [2, 3].

ARID1A is the subunit of the SWI/SNF complex responsible for binding DNA, interacting in a sequence non-specific manner [4]. It is a nucleo-cytoplasmic protein, actively imported from the cytoplasm to the nucleus, where it is accumulated in normal cells [5]. ARID1A expression in the nuclear compartment is observed in normal cells by positive immunohistochemistry (IHC) staining. ARID1A degradation is mediated by the ubiquitin-proteasome system [5].

*ARID1A* acts as a tumor suppressor gene with an epigenetic role in cancer development. Mutations in *ARID1A* occur across the length of the gene and are generally inactivating (frameshift or truncation). They lead to truncated proteins that are rapidly degraded, thus inactivated, resulting in the loss of nuclear protein expression [6]. Loss of ARID1A expression can also depend on the ARID1A promoter hypermethylation [7].

Alterations in *ARID1A* gene occur in about 6% of cancers, including but not limited to clear cell ovarian cancers (45%), endometrial cancers (37%), gastric cancers (20–30%), and bladder cancers (20%) [8, 9].

The mutational profile of ARID1A-mutated cancer was explored in anectodal experiences [9, 10]. The genetic landscape was shown to be different in early stage *vs.* advanced stage cancers, with phosphatidylinositol-4,5-bisphosphate 3-kinase catalytic subunit alpha (*PIK3CA*), phosphatase and tensin homolog (*PTEN*), lysine methyltransferase 2D (*MLL2*), titin (*TTN*) being the most popular concurrent altered genes [9].

Previous research showed that the tumor mutational burden (TMB) of patients with *ARID1A* alterations was significantly higher than in those without, and cancers with multiple *ARID1A* alterations had the highest TMB level [9].

The prognostic impact of ARID1A alterations or expression on cancer patients’ survival remains debated. Loss of ARID1A expression was reported as a favorable prognostic factor in early stage grade 3 endometrioid endometrial carcinoma patients, by Kato et al. [11]. On the contrary, in gastric cancer, loss of ARID1A expression predicts poor overall survival (OS) [12]. In a systematic review and meta-analysis, Luchini et al. [13] reported that loss of ARID1A shortened the time to cancer-specific mortality and to recurrence of cancer.

The predictive role of *ARID1A* alterations after immunotherapy and platinum-based chemotherapy is conflicting. Okamura et al. [14] reported longer progression free survival (PFS) after immune checkpoint inhibitors (ICIs) in ARID1A-mutated cancers [compared to wild-type (WT) tumors], and this result was independent of microsatellite instability or mutational burden. Other two reports suggest the lack of predictive impact of ARID1A for platinum-based chemotherapy and immuno-oncology (IO) therapeutics [15, 16].

ARID1A deficiency was found to sensitize cancer cells to poly adenosine diphosphate (ADP) ribose polymerase (PARP) inhibitors and inhibitors of the DNA damage checkpoint kinase, ataxia telangiectasia and Rad3-related (ATR), providing rationale for clinical testing of PARP and ATR inhibitors [17, 18].

Considering the uncertainties and the current evidence of the literature, we analyzed the data of our population of ARID1A-mutated cancers.

## Materials and methods

Molecular and clinical characteristics of patients evaluated at Phase 1 Unit of Fondazione Policlinico Universitario A. Gemelli IRCCS and performing a genomic analysis were collected. Cancer patients with at least one molecular alteration in *ARID1A* gene were identified as ARID1A^+^. On the contrary, patients with ARID1A WT cancers were identified as ARID1A^–^. The subgroup of ARID1A^+^ patients with a double alteration in ARID1A was identified as ARID1A^2+^. All the molecular analyses were performed using next-generation sequencing (NGS) FoundationOne^®^ CDx. Tissue samples (primary diagnosis or relapsed tissue) were preferentially used for NGS. Blood was used for the analysis if archival tumor tissue was unavailable and a new biopsy was considered unfeasible or not in the interest of the patients by the physician.

Formalin-fixed, paraffin-embedded tumor-containing specimens or blood were sent to the commercial molecular pathology laboratory for NGS in the United States. Details listed in the FoundationOne^®^ reports, obtained by this laboratory, were used for the analysis. Extracted DNA from tumor samples was subjected to NGS utilizing the hybrid capture-based FoundationOne^®^ CDx assay, as previously described [19]. NGS was conducted for exons of 324 genes and introns of 36 genes (FoundationOne^®^ CDx), which are frequently altered in various solid tumors. The indicated genomic regions were investigated for base substitutions, insertions, deletions, copy number variants, rearrangements, microsatellite instability, and TMB.

To be included in the analysis, patients had to sign an informed consent. The study was approved by the institutional research ethics committee.

All the statistical analyses are descriptive and are performed with SPSS v27.0. For all the patients, demographics and data about the disease and the treatment were collected from the health records of the hospital. OS was defined by the time from the date of diagnosis to the date of death. Patients event-free were censored at the date of the last follow-up (i.e. the last date the patient was known to be alive and event-free). Student’s *t* test, Fisher’s exact test, and Mann-Withney test for categorical variables were used, as appropriate. *P* value < 0.05 was considered significant. The median time of survival was calculated and compared using the log-rank test.

## Results

Over a 17 months period, we evaluated, with NGS, 270 patients at our Unit. Twenty-five (9.2%) of them harbored at least one molecular alteration in ARID1A (Table 1).

**Table 1:** Clinical and molecular characteristics of ARID1A^+^ population

**Characteristics**	**ARID1A^+^ population, *n* = 25**	**ARID1A^–^ population, *n* = 245**
Gender		
Female	21 (84%)	172 (70%)
Male	4 (16%)	73 (30%)
Ethnicity		
Caucasic	24 (96%)	239 (97%)
Other	1 (4%)	6 (3%)
Age at diagnosis		
Median, IQR	59 (52–65)	53 (45–61)
Histology		
Gynecological	15 (60%)	83 (34%)
GI	6 (24%)	70 (29%)
Breast	2 (8%)	27 (11%)
Lung	2 (8%)	28 (11%)
Other	0	37 (15%)
Number of metastatic sites		
Median, IQR	2 (1–3)	2 (1–3)
Number of chemotherapy lines		
Median, IQR	2 (1–4)	3 (2–4)
Concurrent medications		
Median, IQR	4 (3–5)	3 (1–5)
CCI		
Median, IQR	10 (8–11)	9 (8–10)
ECOG PS		
0	9 (36%)	101 (41%)
1	14 (56%)	123 (50%)
2	2 (8%)	21 (9%)
Number of mutations		
Median, IQR	6 (5–16)	4 (3–6)
TMB (mut/Mb)		
Median, IQR	20 (10.72–36)	1.26 (0–6)
Number of VUS		
Median, IQR	12 (9–24)	9 (6–12)
Microsatellite status		
MSS	12 (48%)	176 (72%)
MSI-H	8 (32%)	3 (1%)
NA	5 (20%)	66 (27%)
Associated mutations		
PIK3CA	12 (48%)	36 (15%)
TP53	12 (48%)	155 (63%)
PTEN	8 (32%)	15 (6%)
CTNNB1	5 (20%)	11 (5%)
MLL2	5 (20%)	9 (4%)
ASXL1	4 (16%)	3 (1%)

IQR: inter-quartile range; GI: gastro-intestinal; CCI: Charlson comorbidity index; ECOG PS: Eastern Cooperative Oncology Group performance status; mut/Mb: mutations/megabase; VUS: variant of uncertain significance; MSS: microsatellite stability; MSI-H: microsatellite instability-high; NA: not applicable; TP53: tumor protein p53; CTNNB1: catenin beta 1; ASXL1: additional sex combs-like transcriptional regulator 1

### ARID1A^+^ population

Most patients were female (21, 84%) and of Caucasian origin (96%). The median age at diagnosis was 59 (IQR, 52–65) years old. The majority of cancers (60%) were of gynecological origin and diagnosed at an early stage [I to III, tumor-node-metastasis (TNM)]. ECOG PS, at the time of our evaluation, was good (0 or 1) in 92% of the study population although they were pre-treated with a median of 2 lines of chemotherapy.

In half of the population (13, 52%), the molecular analysis was performed on the archival sample referring to the diagnosis time (average time from sample collection 11 months). More than half of the patients with ARID1A^+^ cancer have a high TMB (≥ 20 mut/Mb), with 12 (48%) showing stability of the microsatellite system. PIK3CA, TP53, PTEN, CTNNB1, and MLL2 were the most concurrent mutations (48%, 48%, 32%, 20%, and 20%, respectively) (Table 2).

**Table 2:** Tissue used for NGS analysis in ARID1A^+^ cancers and the most frequent concurrent alterations

**Tissue for NGS**	***PIK3CA***	***TP53***	***PTEN***	***CTNNB1***	***MLL2***
Peritoneum	H1047R; R93Q	-	G165E	-	-
Blood	-	-	E157fs*2	CTNNB1-CTNNB1 deletion	-
Cervix	R93W	R175C	-	-	-
Colon	-	R342*	-	-	-
Lymph node	E545A	-	G132V	S37C	-
Peritoneum	-	K164E	-	-	-
Uterus	-	-	R130G	-	-
Lymph node	-	-	Y27C; R130G	S33P	G1235fs*95
Blood	Splice site 1851-1G>A	-	-	-	P565fs*365
Blood	-	E349*; V216M	-	-	-
Uterus	R38H	R156C	Q87*; D268fs*30	-	-
Ovary	-	R273H	-	-	Q33370fs*22
Cervix	-	I195_L201>M	-	-	-
Peritoneum	-	D281E	-	-	-
Ovary	-	-	-	G34V	-
Colon	E542K; N1044Y	-	-	-	-
Ovary	E542K	-	-	-	-
Stomach	H1047R	K382fs*40	-	-	-
Breast	E365K; E453K	-	-	-	G3465fs*37; A2205fs*59
Duodenum	-	R248W	-	-	-
Soft tissue-bone	-	-	-	-	-
Pancreas	R108H	-	-	N387Y	P2354fs*30
Lymph node	R108H; R88Q	P152L; V73fs*50	R130G	-	-
Uterus	H1047R	P301fs*44	R130G; splice site 634+5G>A	-	-

-: none

Endometrioid cancer of endometrium was the prevalent tumor, representing 24% of the cases, followed by clear cell ovarian cancer (5, 20%). Among the pathogenic molecular alterations in ARID1A, frameshift alterations were the most common (19/31, 61%) (Table 3). Interestingly, all the patients displayed different alterations apart from the Q372fs*19 that was shared by 3 patients with different histologies (1 endometrioid endometrial, 1 endometrial not-endometrioid, 1 clear cell ovarian).

**Table 3:** Molecular alterations in ARID1A^+^ tumors

**Tissue used for analysis**	**Molecular alterations in ARID1A**	**Hystology**
Primary disease (diagnosis)	ARID1A-PTAR1 truncation, S2249*	Ovarian (clear cell)
	L1731fs*4; Q372fs*19	Endometrial (endometrioid)
	N104fs*7	Cervical
	P1175fs*5; Q766fs*67	Endometrial (endometrioid)
	Q372fs*19	Ovarian (clear cell)
	R727fs*84	Ovarian (clear cell)
	W2049*	Colorectal
	P224fs*8	Gastric
	Y2254*	Cervical (clear cell)
	S1171fs*22	Duodenal
	ARID1A truncation	Lung
	P146fs*86	Pancreas
	K1072fs*21-subclonal	Endometrial (endometrioid)
Relapsed disease	Q1409*; S1609fs*38	Endometrial (endometrioid)
	Q1519fs*8; G623fs*6	Endometrial (endometrioid)
	Q372fs*19	Endometrial
	Q567*	Ovarian (clear cell)
	W1073fs*32	Breast
	Y447*	Cervical
	Y788*	Ovarian (high grade)
	F2141fs*59, M1564fs*1	Endometrial (endometrioid)
Blood	ARID1A-ARHGEF40 truncation	Hepatocellular
	Q1327fs*11	Colorectal
	Q1579*	Breast
	Q754*	Lung

PTAR1: prenyltransferase alpha subunit repeat containing 1; ARHGEF40: Rho guanine nucleotide exchange factors gene 40

### Comparison with mutation landscape in ARID1A^–^ population

**Figure 1:**
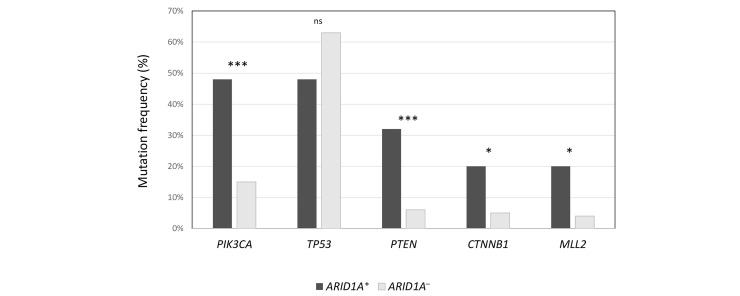
Frequency of the most common concurrent mutations in ARID1A^+^ population, compared to ARID1A^–^ cancers. ^***^ *P* < 0.0001; ^*^ *P* < 0.01; ns: not significant

The median number of mutations in ARID1A^+^ population was significantly higher compared to ARID1A^–^ population (6 *vs.* 4, *P* = 0.02), as well as TMB (20 *vs.* 1.26, *P* < 0.00001). Also, the median number of VUS was higher in ARID1A^+^ cancers (12 *vs.* 9, *P* = 0.002). In Figure 1, the authors show the enrichment of some alterations (*PIK3CA*, *PTEN*, *CTNNB1*, and *MLL2*) in ARID1A^+^ population. On the contrary, *TP53* was prevalent in ARID1A^–^ population (63% *vs.* 48%), although not statistically significant.

Comparing the response to platinum for gynecologic patients with ARID1A^+^ tumors *vs.* ARID1A^–^ tumors, the presence of alterations did not relate with a different time to platinum treatment failure (16 months *vs.* 17 months, respectively). OS for ARID1A^+^ patients was 66 months.

### ARID1A^2+^ population

Interestingly, the authors found six patients with 2 different alterations in ARID1A^+^ (ARID1A^2+^). They were all women with gynecological cancer, mainly of endometrial origin. Five (83%) had endometrioid endometrial cancer and the other one was diagnosed with clear cell ovarian cancer. ECOG PS was 0 or 1. TMB was 24.5 mut/Mb, the higher found among the ARID1A^+^ patients, with the majority (4, 67%) displaying microsatellite instability.

The response to immunotherapy of the endometrial cancer patients was variable: two patients with high TMB (48/20 mut/Mb) and MSI-H progressed within 6 months; other two patients with MSI-H and TMB intermediate/high (14/29 mut/Mb) reached 14 months and 27 months of PFS, with one of them being still on treatment. The last patient with TMB low/MSS progressed after two cycles of immunotherapy. To note that one patient with MSI-H/high TMB endometrioid endometrial carcinoma progressed on immunotherapy very fast but showed a partial response to PI3K inhibitor lasting for 7 months.

## Discussion

ARID1A is being configured as a potential new target for cancer treatments. A variety of compounds are being tested in ARID1A^+^ cancers, without any drug approval so far. These medicines include immune checkpoint blockades, PI3K/AKT/mTOR inhibitors, PARP inhibitors, ATR inhibitors, enhancer of zeste homolog 2 (EZH2) inhibitors, and pan-histone deacetylase (HDAC) inhibitors. In Table 4, it reported ongoing clinical trials for ARID1A-mutated cancers [20].

**Table 4:** Ongoing clinical trials in ARID1A-mutated cancers

**Clinical trial number**	**Phase**	**Randomization**	**Disease**	**Setting**	**Experimental arm**	**Control arm**	**Primary objective**	**Status**
NCT05023655	2	No	Solid tumor	Advanced	Tazemetostat	NA	ORR	Recruiting
NCT04284202	2	No	NSCLC	Advanced	PD-1 + dasatinib	NA	PFS	Unknown
NCT04065269	2	No	Ovarian and endometrial clear cell carcinoma	Advanced	AZD6738 +/– olaparib	NA	ORR	Recruiting
NCT04953104	2	No	Urothelial cancer	Advanced	Nivolumab	NA	ORR	Not yet recruiting
NCT04957615	2	No	Solid tumor	Advanced	Nivolumab	NA	ORR	Recruiting
NCT05154994	1	No	Urothelial cancer	Advanced	Tremelimumab + durvalumab + belinostat	NA	RP2D	Recruiting
NCT04633902	2	No	Melanoma	Advanced	Olaparib + pembrolizumab	NA	ORR	Recruiting
NCT03682289	2	No	Solid tumor	Advanced	AZD6738 +/– olaparib	NA	ORR	Recruiting
NCT02576444	2	No	Solid tumor	Advanced	AZD5363 + olaparib	NA	ORR	Active, not recruiting
NCT05379972	2	No	Gastric cancer	Advanced	Pembrolizumab + olaparib + SBRT	NA	ORR	Not yet recruiting
NCT03207347	2	No	Solid tumor	Advanced	Niraparib	NA	ORR	Active, not recruiting
NCT04042831	2	No	Biliary tract cancer	Advanced	Olaparib	NA	ORR	Recruiting
NCT04104776	1-2	No	Solid tumor, lymphoma	Advanced	CPI-0209	NA	DLTs; ORR	Recruiting

ORR: overall response rate; NSCLC: non-small cell lung cancer; PD-1: programmed death-1; RP2D: recommended phase 2 dose; SBRT: stereotactic body radiation therapy; AZD6738: ceralasertib; CPI-0209: tulmimetostat; DLTs: dose-limiting toxicities

In particular, a significant interest in this gene is rising in the gynecological field, due to the high percentage of ARID1A alterations among these patients. For instance, ATARI trial is a multicenter, international, phase II study (NCT0405269) testing the ATR inhibitor ceralasertib as a single agent and in combination with olaparib in ARID1A gynecological cancers. *ARID1A* deficiency was defined as the absence of tumor expression of its gene product (BAF250a) by IHC staining. Of note, tumors harboring pathogenic mutations in ARID1A do not necessarily have a loss of expression of BAF250a, which was reported in 67% of the study population [21]. The interim analysis in ARID1A-deficient tumors showed an ORR of 20%, with two complete responses among the 10 enrolled patients [21].

A deeper insight into the clinical and molecular landscape of ARID1A^+^ tumors may guide the design of future clinical trials targeting this gene. Therefore, we retrospectively analyzed our cancer population undergoing molecular evaluation with FoundationOne^®^ CDx assay.

In the population of patients being offered an NGS test for their advanced tumor, we found 9% of ARID1A^+^ cancers, a percentage comparable to that reported in the literature (6%) [8]. The slightly higher numbers are due to the enrichment in gynecological cancers, for whom we are a referral center in Italy. According to previous analyses [6, 8], endometrial cancers of endometrioid subtype and clear cell ovarian cancer were the most represented tumors, accounting for 44% of our study population. A higher mutational load (TMB, VUS number, and co-occurrent mutations number) was observed in ARID1A^+^ population, especially in the ARID1A^2+^ group, whose median TMB reached 24.5 mut/Mb. *PIK3CA*, *TP53*, and *PTEN* were the most common concurrent mutations in ARID1A^+^ population, although *TP53* was slightly increased in ARID1A^–^ cancers (63% *vs.* 48%).

Previous evidence suggested that ARID1A inactivation alone is insufficient to drive ovarian tumorigenesis [22]. Defects in *PIK3CA* or *PTEN*, frequently co-occurring, may cooperate with ARID1A loss to drive cancer and downstream activation of the PI3K/AKT pathway [22]. Moreover, ARID1A mutations often occur in tumors of specific subtypes (i.e. clear cell ovarian carcinoma), refractory to chemotherapy. ARID1A-deficient cancer cells demonstrate increased sensitivity to treatment with small molecule inhibitors of the PI3K/AKT pathway [23].

In a previous experience with GI cancers [10], *TP53*, Kirsten rat sarcoma viral oncogene homologue (*KRAS*), and adenomatous polyposis coli (*APC*) occurred most frequently in ARID1A^+^ GI cancers [10]. This observation may support the idea that the presence of different concurrent mutations might be influenced by the histotype of the primitive cancers and the site of disease, rather than a particular pathway guided by or concurrent with ARID1A.

In our experience, ARID1A alterations were found both in solid tissue (primary diagnosis and relapsed disease) and blood. A recent report among 71,301 patients suggests that liquid biopsies identified ARID1A alterations at a frequency similar to that found in primary tumor material [24]. If mutations in ARID1A are present in the primary tumor such as atypical meningiomas, these tumors tend to have a worse prognosis [25].

Double alterations in ARID1A were observed only in solid tissues. Probably, it may be linked to the absence of gynecological cancers, the ones identified as ARID1A^2+^, among the patients who underwent liquid biopsy.

With the limitation of the small sample size, our study did not show any differences in terms of duration of response to platinum-based chemotherapy among gynecological cancer displaying ARID1A alterations as compared with ARID1A^–^ tumors. Moreover, we observed a poor outcome among ARID1A^+^ tumors compared to the ARID1A WT cohort, consistently the largest report so far [13].

It is uncertain if monoallelic inactivations share the same phenotype (i.e. loss of ARID1A expression) as biallelic inactivations [5]. This analysis lacks information about ARID1A expression by IHC and the match between molecular alterations (mono or biallelic) and IHC (presence, total or partial loss of the protein).

In conclusion, this analysis confirms and strengthens existing literature (pretty high frequency of ARID1A alterations among solid tumors, especially particular subtypes of gynecological cancers, higher value of TMB, poorest outcomes among ARID1A^+^ cancers, no predictive role for platinum-based chemotherapy). In addition, we provide clinical and molecular details in both ARID1A^+^ and ARID1A^2+^ cohorts, highlighting Q372fs*19 as the most frequent alteration in solid tissue.

Due to the high frequency of concurrent alterations in ARID1A and PI3K pathway in some subtypes of gynecological cancers (i.e. endometriod endometrial carcinoma, clear cell ovarian cancer), we propose to combine ATR inhibitor or PARP inhibitor with PIK3CA inhibitors in a new trial to test the synergic action of these drugs. Genomic analysis and protein expression of cancer tissue, as well as liquid biopsy, need to be performed and correlated with response to therapy.

The identification of *ARID1A* among the key genes mutated in clear cell ovarian cancer may offer new treatment opportunities in this orphan disease, poorly responsive to chemotherapy. Moreover, among ARID1A^2+^ endometrioid endometrial carcinomas with a high value of TMB, a combination or new immunotherapies may be proposed in future studies.

## Abbreviations

AKT: protein kinase B
ARID1A: AT-rich interaction domain 1A
ATR: ataxia telangiectasia and Rad3-related
BAF250a: Brahma-related associated factor 250a
CTNNB1: catenin beta 1
ECOG PS: Eastern Cooperative Oncology Group performance status
GI: gastro-intestinal
IHC: immunohistochemistry
IQR: inter-quartile range
MLL2: lysine methyltransferase 2D
MSI-H: microsatellite instability-high
mut/Mb: mutations/megabase
NGS: next-generation sequencing
ORR: overall response rate
OS: overall survival
PARP: poly adenosine diphosphate ribose polymerase
PFS: progression free survival
PI3K: phosphoinositide 3-kinase
PIK3CA: phosphatidylinositol-4,5-bisphosphate 3-kinase catalytic subunit alpha
PTEN: phosphatase and tensin homolog
TMB: tumor mutational burden
TP53: tumor protein p53
VUS: variant of uncertain significance
WT: wild-type

## Declarations

### Availability of data and materials

The data generated in the present study can be requested from the corresponding author (Gennaro Daniele, gennaro.daniele@policlinicogemelli.it).

### Copyright

© The Author(s) 2023.
